# *Ex vivo* determination of bone tissue strains for an *in vivo* mouse tibial loading model

**DOI:** 10.1016/j.jbiomech.2014.03.035

**Published:** 2014-07-18

**Authors:** Alessandra Carriero, Lisa Abela, Andrew A. Pitsillides, Sandra J. Shefelbine

**Affiliations:** a Department of Bioengineering, Imperial College London, UK; b Department of Veterinary Basic Sciences, Royal Veterinary College, London, UK

**Keywords:** Bone strain, Digital image correlation, Mouse, Loading, Tibia

## Abstract

Previous studies introduced the digital image correlation (DIC) as a viable technique for measuring bone strain during loading. In this study, we investigated the sensitivity of a DIC system in determining surface strains in a mouse tibia while loaded in compression through the knee joint. Specifically, we examined the effect of speckle distribution, facet size and overlap, initial vertical alignment of the bone into the loading cups, rotation with respect to cameras, and *ex vivo* loading configurations on the strain contour maps measured with a DIC system.

We loaded tibiae of C57BL/6 mice (12 and 18 weeks old male) up to 12 N at 8 N/min. Images of speckles on the bone surface were recorded at 1 N intervals and DIC was used to compute strains. Results showed that speckles must have the correct size and density with respect to the facet size of choice for the strain distribution to be computed and reproducible. Initial alignment of the bone within the loading cups does not influence the strain distribution measured during peak loading, but bones must be placed in front of the camera with the same orientation in order for strains to be comparable. Finally, the *ex vivo* loading configurations with the tibia attached to the entire mouse, or to the femur and foot, or only to the foot, showed different strain contour maps.

This work provides a better understanding of parameters affecting full field strain measurements from DIC in *ex vivo* murine tibial loading tests.

## 1 Introduction

Bones adapt their architecture in response to the applied loads. However, it is still unclear how bone mechano-adaptation is spatially coordinated. In most of the animal bone adaptation models, it is assumed that adaptation occurs in response to a peak strain stimulus (Rubin and Lanyon 1984, Gross et al., 1997, Judex et al., 1997, Mosley et al., 1997, Forwood et al., 1998, Mosley and Lanyon 1998, Cullen et al., 2001, Robling et al., 2001, Lee et al., 2002, Mosley and Lanyon 2002, De Souza et al., 2005, LaMothe et al., 2005). While loads are applied *in vivo* on bone, these models have all required pre-calibration in order that the applied strains can be controlled. These studies have thus often related mechano-adaptation of bone to the strain distributions that estimated either experimentally on the basis of measurements made only at single locations of the bone surface using strain gauges *in vivo,* or in representative *ex vivo* loading. Other times, strains have also been estimated computationally with finite element analysis, which requires that assumptions are made regarding the material properties, geometry, and loading conditions. These methods are clearly limited in their scope to provide direct measurements of strain across an entire bone surface and so more recently, digital image correlation (DIC) has been used to overcome these limitations offering a viable method for measuring high resolution full-field strains on the whole bone surface (Sztefek et al., 2010). Although being an *ex vivo* technique, DIC is particularly suitable for biological applications, as it reveals strains in inhomogeneous, anisotropic and non-linear materials, like bone, which has also a complex morphology (Nicolella et al., 2001, Verhulp et al., 2004, Nicolella et al., 2005, Hoc et al., 2006, Yang et al., 2007, Sztefek et al., 2010, Yamaguchi et al., 2011). Furthermore, DIC provides only surface strain measurements and not volumetric measurements as FE models. However, this is sufficient for animal models of cortical bone adaptation, as bone formation occurs mainly on the periosteal surface (Sztefek et al., 2010).

In DIC, the surface of the sample of interest is speckled with high contrast and observed by the charge-coupled device (CCD) cameras while loaded. The field of view is then divided into subsets called ‘facets’, which are unique correlation areas containing a specific number of pixels, and the displacement is computed by tracking the relative movements between the facets. The facets track features of speckles during loading and measure their deformation. A large facet size improves the accuracy of measurements, but a small facet reduces the computation time and captures local effects. The facets overlap each other ensuring continuity of strain across facets. A large overlap increases the computational time for calculating strains and can result in redundant measurements. The final result is the measurement of the axial, transverse, and shear surface strain.

We have previously used DIC to determine surface strains in mouse tibiae while loaded in compression through the knee joint (Sztefek et al., 2010). We showed that bone in the ‘adapted leg’ (six bouts of loading over two weeks) had lower strains compared to the contralateral leg. This study demonstrated that DIC was able to determine local strains over the entire surface of the bone under *ex vivo* testing conditions.

Here we test how sensitive DIC strain measurements are to the test set-up. In this study we generate 3D contour maps of the strain fields on the bone surface during mouse tibia loading (De Souza et al., 2005) and determine the sensitivity of the DIC method to different speckles, facet sizes and overlaps, positioning of the bone within the loading cups and rotation of the bone in front of the camera. Furthermore, we investigate the effect of different *ex vivo* bone loading configurations (whole mouse, disarticulated hind limb, or lower limb) on the mouse tibial surface strains.

## 2 Material and methods

Fresh frozen male C57BL6 mice (Charles River Company, UK) at either 12 or 18 weeks of age (w.o.) were used in this study. Mice were defrosted and soft tissue was removed from both their tibiae. Each tibia was covered with a thin layer of matt, water-based, white paint (Dupli-Color Aqua Lackspray, Motip Dupli GmbH, Germany), and then speckled with matt, acrylic, black ink (Acrylic Ink, Daler Rowney, England) using a high precision air brush (SprayCraft SP50K, Shesto, England). We determined the optimal size of speckles (see below).

**Fig. 1:**
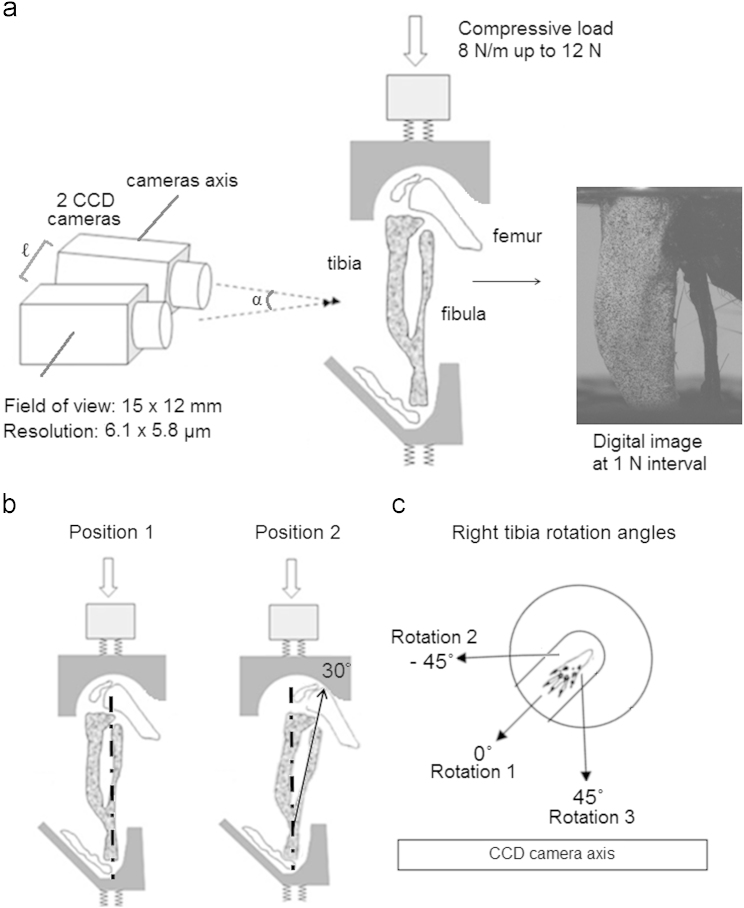
(a) DIC set-up used to obtain images of the medial side of the tibia (Adapted from (Sztefek et al., 2010)), (b) diagrams showing the two loading positions tested. In *Position 1* the bone is axially loaded, whereas in *Position 2* the bone is loaded at a 30° flexure respect to the vertical axis, and (c) transversal view of the bottom loading cup showing the position of the mouse foot (the top cup at the knee was also rotated at the same amount) when the right tibia was loaded at 0°, −45° and +45° rotation angle with respect to the CCD camera axis. *Rotation 1* refers to the position of the medial surface of the tibia when parallel to cameras, *Rotation 2* refers to the position of the medial surface of the tibia at −45° with respect to cameras and finally *Rotation 3* refers to the position of the medial surface of the tibia at 45° respect to the camera axis.

Right and left tibiae of each mouse were speckled together to ensure similar speckle distributions. To measure strain during the application of load, limbs were loaded at a rate of 8 N/min up to 12 N with 50 N load cell (Instron 5866, High Wycombe, UK) using custom built loading cups, which applied an axial load on the tibia across the knee and ankle joints (De Souza et al., 2005). 12 N is not a physiological load for the mouse knee, but refers to a load that has been used when investigating cortical bone mechanoadaptation in animal models (De Souza et al., 2005). Two CCD cameras (50 mm lenses with 20 mm distance rings, GOM GmbH, Germany) mounted on a tripod were positioned horizontally in front of the loading fixture, at a distance of 20.5 cm, to provide a 15 mm×12 mm field of view (6.1 µm x 5.8 µm resolution) with the depth of focus field of 0.9 mm (Fig. 1a). The two cameras were separated by 36 mm along their axis (length *ℓ*, Fig. 1a) and they were rotated towards each other meeting at 25° angle (angle α, Fig. 1a) on the bone surface. Calibration was conducted by using a high-precision 15 mm×12 mm panel (GOM GmbH, Germany). A pair of light-emitting diode lamps, with polarised filters, were set to illuminate the specimen and boost the visibility of its surface speckle pattern. Only the medial surface of the tibiae was imaged as the two camera system allowed the view of only one bone surface. Images were collected at each 1 N interval (with a frame rate of about 0.133 Hz), and post-processing software (ARAMIS 5 M System, GOM GmbH, Germany) was used to calculate strains on the bone surface (Fig. 1a). Processing of images involved a sensitivity study to define the ideal facet size and overlap (see below).

Preceding the actual measurement of load-induced strains on the bone surface, three images of the non-loaded tibia surface were captured in order to evaluate the amount of experimental noise (accuracy) generated by the camera system during image acquisition. Load-induced strains were measured twice on each tibia to ensure reproducibility of measurements. Maximum and mean strains on the surface were calculated using the linear strain computation algorithm of ARAMIS. This uses the displacement of the data points (centres of the facets) to calculate the strain. All strains were computed with a computation size of 5, i.e. a 5×5 field of 3D points was used to calculate strain value at the facet centre, and a validity quote of 65%, i.e. at least 16 of the 25 data points (for a field of 5×5) around the facet centre must exist for the strain to be computed at this location.

All bones were kept wet before and between measurements by wrapping them in saline moistened gauze.

### 2.1 Speckle distribution

The dependence of the strain map on speckle distribution and size was tested using three different speckle patterns consecutively on the left and right tibia of three 18 w.o. mice. Specifically, *Speckle 1* had dots of ~4 pixels in diameter with 40% black/white density, *Speckle* 2 had dots of ~8 pixels in diameter with 45% black/white density and *Speckle 3* had dots of ~12 pixels in diameter with 55% black/white density. These three speckle types were produced by the same ink and high precision air brush, by varying the aperture of the air brush nozzle. The images were analysed by using the facet size (19×19) and overlap (21%) proposed as default by the DIC system. To define which speckle pattern gives the most accurate results with our set-up, we compared the noise generated by speckles at zero loading. As a result from this test *Speckle 2* was applied to all the following tests.

### 2.2 Facet size and overlapping

During processing of images, we compared strain contour maps of six tibiae (12 w.o.) calculated using three quadrangular facet sizes of 15×15, 19×19 and 20×20 pixels with two overlaps of 5–7% and 20–21%. Noise at zero load, and maximum and average strains for four different load cases (3, 6, 9 and 12 N) were calculated. As a result from this test facet size 19×19 with overlap 20% was used to process images of all the following tests.

### 2.3 Right and left leg

Because cups were originally designed to allow loading of the right leg, we compared DIC results captured from loaded right and left legs of six 18 w.o. mice in order to test whether any difference in strains engendered could be detected.

### 2.4 Precision of measurement on a single bone

A series of 10 consecutive tests were carried out on the right tibia of one 18 w.o. mouse and the strain distribution for each test was recorded, maintaining all parameters constant, to determine repeatability in measuring strains on a single mouse bone. Between each test, the specimen was not removed from the loading cups, but the load was completely removed. The time between each load was 5 min, and the bone was kept moist throughout the testing.

### 2.5 Loading positions within the cups

The loading cups used here were specifically designed to load the tibia axially (De Souza et al., 2005). Tests conducted on the right and left tibiae of three 18 w.o. mice were performed to investigate the difference in strain patterns when the tibia was loaded axially (*Position 1*, Fig. 1b) and with the tibia positioned at 30° with respect to the vertical axis (*Position 2*, Fig. 1b). *Position 2* was achieved by locating the knee joint at the edge of the notch within the cup and measured by the angle of the fibula in the first picture of the bone under static condition.

### 2.6 Rotations in front of the cameras

Sensitivity of the strain map to the rotation of the surface of interest in front of cameras was also measured. Strains from both the right and left tibiae of two 18 w.o. mice were computed when the bone was loaded with its medial surface parallel to cameras (*Rotation 1*, Fig. 1c) and also when at an angle between −45° and +45° with respect to the camera plane (*Rotations 2* and *3*, Fig. 1c, respectively).

### 2.7 Loading configurations

Right and left tibiae from three 12 w.o. mice were loaded in three different *ex vivo* loading configurations: (1) with the entire mouse body attached, (2) with only the foot and the femur and its muscles attached, and finally (3) with the tibia and foot alone.

### 2.8 Statistical analysis

Normal distribution of the maximum and average strains and their homogeneity of variance were analysed by the Shapiro–Wilk test and Levene׳s test, respectively (SPSS, IBM, Somers, NY). Difference in maximum and average strains between left and right legs, and between *Position 1* and *Position 2*, were analysed with a *t*-test. Difference in strain values between three speckles and three rotations the three loading configurations were compared using analysis of variance (one-way independent ANOVA) and Post Hoc procedures for multiple comparisons. All tests were two tailed and *p*-values smaller than 0.05 were considered to be significant.

Precision error (PE) of measurement on a single bone was estimated in terms of coefficient of variation (CV) of repeated measurements given in percentage (Glüer et al., 1995).

## 3 Results

The spatial strain distribution over the medial side of the tibia was calculated, based on the three components of displacement measured by the DIC system. The results reported here show the strain in the axial (loading) direction as transverse strain and shear strain had relatively low magnitude compared to the axial strain.

**Fig. 2:**
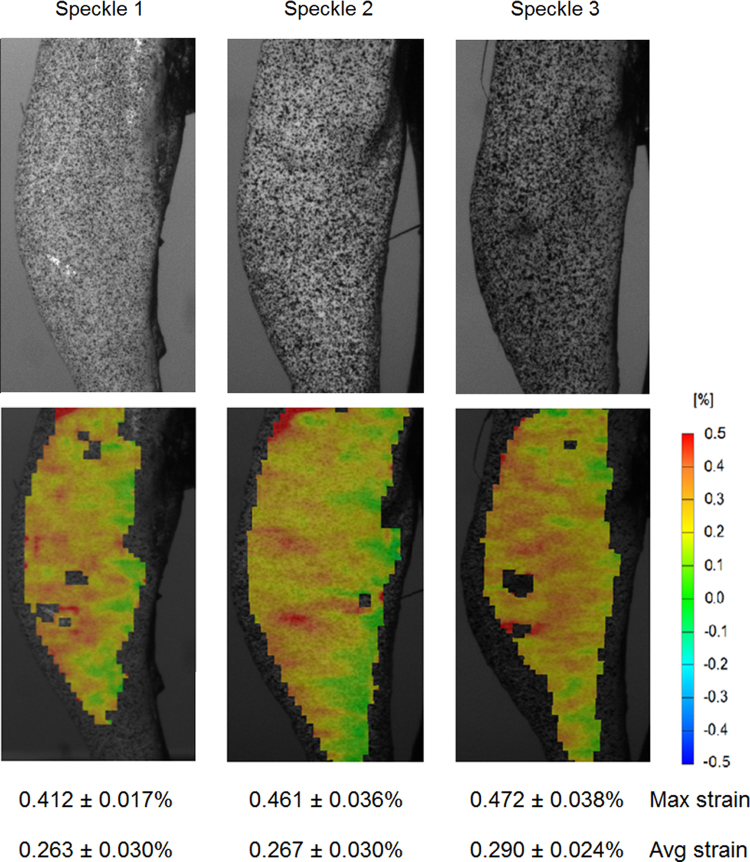
Strain contour maps at 12 N for three different speckle patterns on the same tibia from an 18 w.o. mouse.

Tibial axial compression of 12 N results in tension on the antero-medial side of the tibia (Fig. 2) with a peak tensile strain of up to ~0.5% (5000 microstrain) reflecting the naturally curved morphology of the tibia. Similar load-deformation curves were observed during each loading episode conducted on the same age mice under the same test conditions, indicating that there was no bone failure during the loading.

### 3.1 Speckle distribution

Results from the speckle dependence test showed that the average strain distributions obtained over the medial tibial surface from three different speckles were similar (Fig. 2). However, for *Speckle 3* the tensile strain peak was significantly higher (*p*<0.005) than in *Speckle 1* (0.472±0.038% in *Speckle 3* vs. 0.412±0.017% in *Speckle 1)* probably due to an increase in the speckle density in the specific location (Fig. 2). *Speckles 1* and *3* also had facets missing strain calculation, due to the low and high speckle size with respect to the chosen facet size. Noise at zero strain showed that *Speckle 2* (0.049±0.004) generated statistically significant smaller (*p*<0.05) noise than *Speckle 1* (0.040±0.005), but was similar noise to *Speckle 3* (0.045±0.008).

### 3.2 Facet size and overlapping

**Fig. 3:**
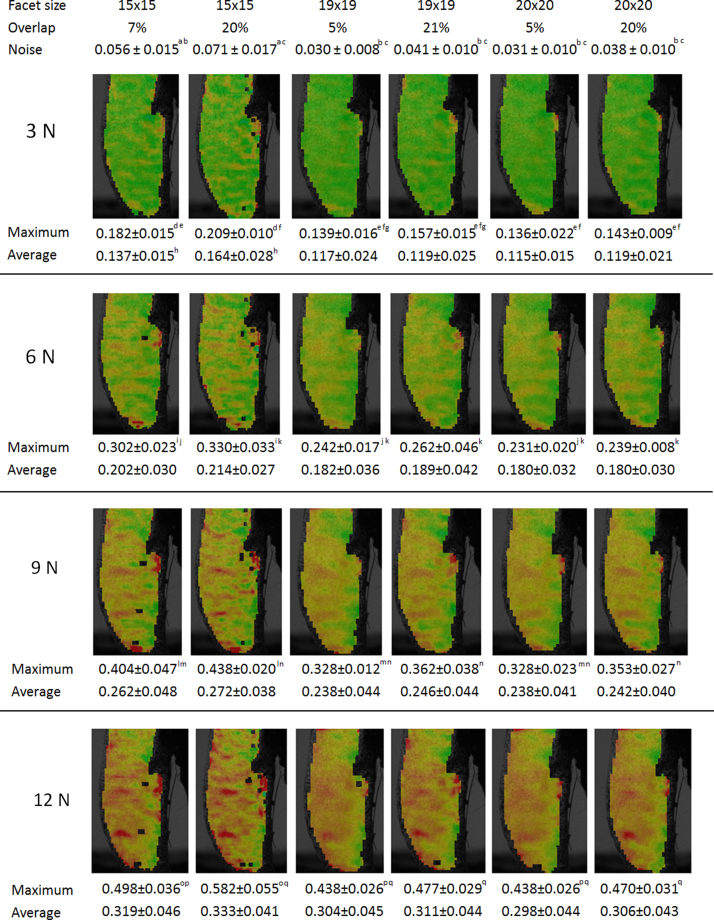
Strain map on the bone surface with maximum and average values at four loading instances (3, 6, 9 and 12 N) when different facet sizes and overlaps were considered. The letters (a–q) indicate a statistical significant difference (*p*<0.05) between the variables calculated with different computational analysis when the same load is applied.

Overlay plots displaying strains at four different stages of the loading (Fig. 3) show that the studied area is covered similarly with very similar strain values and patterns when larger facet sizes are used. When the smallest facet size is used instead there are missing parts and maximum strain is always higher than in the large facets. Using the biggest facet size of 20 pixels, the strain pattern was smooth, compared to the 19 pixels facet size and a 20% overlap, where more features on the high deformation zone were captured. When the facet size was decreased further to 15 pixels, new details were revealed but the accuracy decreased (noise increased), rendering this computation unfavourable compared to the other two.

### 3.3 Right and left leg

When comparing left and right leg, we observed a very similar strain distributions, peak and average strain magnitude on the medial surface of two tibiae with a maximum and average surface strain respectively of 0.454±0.054% and 0.261±0.038% for the left leg, and 0.465±0.044% and 0.255±0.033% for the right leg (*p*=0.481 and 0.387, for maximum and average strain, respectively).

### 3.4 Precision of measurement on a single bone

When ten consecutive tests were repeated on the same bone, strain distribution was repeatable with the peak strain always located on the medial mid-diaphysis surface of the bone (maximum strain=0.453%±0.028%, average strain=0.260%±0.014%, average noise=0.035±0.009). The PE_CV was equal to 6.15% for the maximum strain and to 5.38% for the average strain.

### 3.5 Loading positions within the cups

Strain distributions obtained for tibiae loaded in *Position 1* and *Position 2* within cups were similar at 12 N, with a maximum and average surface strain respectively of 0.460±0.031% and 0.262±0.026% when the tibia was loaded in *Position 1*, and 0.456±0.028% and 0.283±0.021% when loaded in *Position 2* (*p*=0.877 and 0.204, for maximum and average strain, respectively). The tibia initially loaded in *Position 2* almost completely self-aligned to *Position 1* while loaded up to 12 N.

### 3.6 Rotations in front of the cameras

**Fig. 4:**
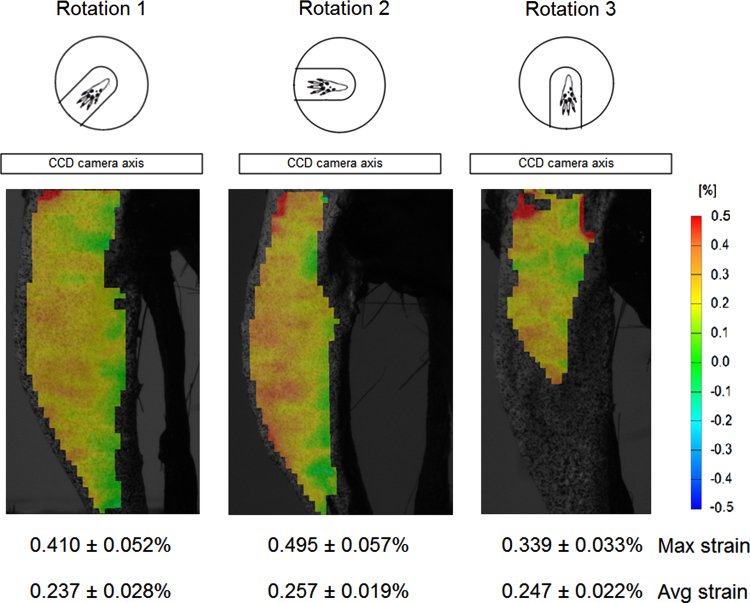
Strain distributions obtained at 12 N for the same mouse tibia positioned with the medial surface of the bone parallel to cameras (*Rotation 1*) and at −45° and 45° of rotation (*Rotation 2* and *Rotation 3*, respectively).

Strain distributions differed when the tibiae were rotated with respect to the camera plane. When bone was positioned and loaded in *Rotation 1*, maximum and average strains were 0.410±0.052% and 0.237±0.028%, respectively. Higher peak strain magnitudes (*p*<0.05) were obtained when the bone surface was rotated at 45° to the camera axis (*Rotation 2*), with a maximum surface strain of 0.495±0.057% (average strain was 0.257±0.019%) (Fig. 4). When bone was positioned in *Rotation 3*, at −45° in front of cameras, only a partial surface strain map was detected due to the curvature of the bone surface. The location of the peak strain was not detected in this configuration so that maximum strains of the computed bone surface strain were markedly smaller (0.339±0.033%, average strain was higher 0.247±0.022%) than in tibiae loaded with cameras in *Rotation 1* and *2* (*p*<0.05 and *p*<0.005, respectively).

### 3.7 Loading configurations

**Fig. 5:**
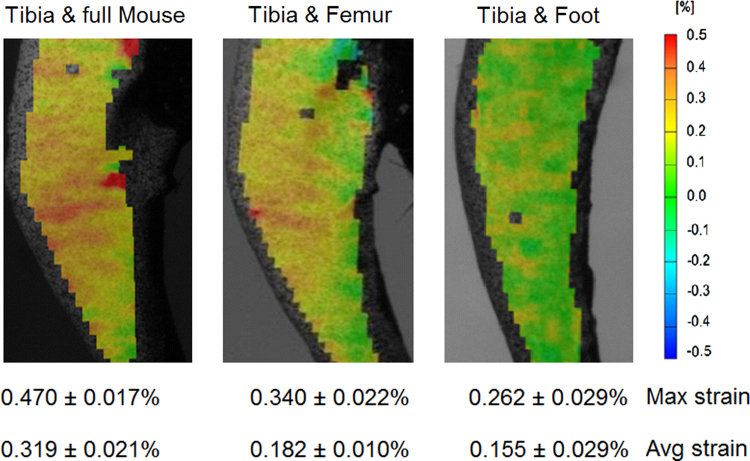
Strain maps on the medial surface of the right tibia of a C57BL/6 mouse, 12 w.o. male, obtained at 12 N when loaded in three different configurations (i.e. with the entire mouse, with only the femur and foot, and with only the foot attached to the tibia).

Local regions of high strain (0.470±0.017%) were seen on the medial side of all tibiae loaded with the full mouse body, but these levels of strain were only very rarely observed when tibiae were loaded in either of the two other loading configurations (Fig. 5). Across the whole bone, the maximum strain significantly (*p*<0.001) decreased when only the femur and its soft tissues were still attached (0.340±0.022%) and decreased (*p*<0.001) even more markedly with the tibia and foot alone (0.262±0.029%); peak strains in the latter two configurations also differed significantly (*p*<0.001) from each other. Furthermore, the average strain level across the entire surface was significantly higher (*p*<0.001) when the tibia was loaded in the full mouse configuration (0.319±0.021%) compared to either of the other two (femur: 0.182±0.010%, foot: 0.155±0.029%) configurations, which did not differ from each other. The load-deformation curve was similar for three loading conditions, indicating that there was no failure during the load.

## 4 Discussion

In this study we demonstrate the sensitivity of DIC strain measurements on the murine medial tibial surface to different testing parameters during loading. Although only 4–6 bones were tested for each sample group, we were able to detect statistical significant differences in variables investigated, offering an overview of parameters affecting full field strain measurements in *ex vivo* murine tibial loading tests. A summary of tests conducted with respective results is shown in Table 1.

Maintaining the correct speckle size and density is extremely important for DIC analysis. When using a 19×19 facet size, ideal speckles would be of ~8 pixels in diameter with a 60–40 ratio between background and speckles for the study of a mouse bone of this size. Our study showed that when using a speckle of ~4 pixels in diameter with a lower density (*Speckle 1*) as well as ~12 pixels in diameter with a higher density (*Speckle 3*), it was not possible to calculate the strain in all facets on the bone surface. This is in agreement with previous studies, which have indicated that a speckle with less than 4 pixels in diameter is not recognisable by the DIC system (Lecompte et al., 2006). Pixels that are too small or too big reduce the contrast of the image within the single facet so strain is not computed. To simplify sample preparation and render it independently of the user, previous studies (Lecompte et al., 2006, Haddadi and Belhabib 2008) suggested printing an optimised speckle pattern on the specimen relative to its size, but this becomes obviously very difficult on small and irregular surface of biological samples such as mouse tibia. Accordingly, the use of a high-precision paint brush remains the most accurate way to control the speckle size on mouse bones.

The same speckle computed with three different facet sizes, showed minor variability between strains distributions of the large facet sizes. However, strain calculation was not computed in some local parts of the bone when using the smaller facet size (15×15) and noise increased. This means that large speckles require larger facets in order to be computed because otherwise it would not be possible to detect contrast in the specific areas. The optimal facet size and overlap will depend on speckle size and density and vice versa. Therefore the best post-processing settings should be identified for each sample tested. Our results suggest that for a mouse tibia bone a ratio of 1:2 should be kept between speckles diameter and facets size (i.e. speckles of ~8 pixels diameter and facet size of 19×19 pixels) for the strain to be computed.

Initial alignment of the tibia into the loading cups did not affect the surface strain at 12 N as the compressive force acting on the knee and ankle joints causes an automatic adjustment during loading of the knee to the recommended axial loading position (i.e. *Position 1*). Furthermore, when the full mouse was attached to the tibia, it was not possible to load the leg within cups in any other position than the two explored. This indicates that this load set-up together with the shape of cups make this particular system relatively insensitive to how the knee is originally placed in cups, as the tibia will align itself during compression.

Rotation of the tibia in front of the camera affected the strain distribution on bone, with an increase in the strain magnitude when bone surface was not parallel to cameras (as shown in the −45° rotation). When rotated with respect to the camera plane, speckles acquire a different shape and size as the bone surface was closer or farther from cameras. If the surface is curved, rotation with respect to cameras may also make it impossible for the cameras to see the whole surface and data will be lost (as shown in the +45° rotation). For reproducible and accurate results, the imaged surface should be parallel to the camera plane.

*Ex vivo* testing is often performed to estimate *in vivo* conditions. Here we show that disarticulation of the limb significantly affects results (Table 1). If accurate strain magnitudes are required, it is critical that the in vivo loading conditions are replicated as closely as possible. However, if strain patterns are more of interest than strain magnitudes, comparisons can still be made across bones with a consistent tibia–femur set-up.

The loading cups we used for our study were initially developed for *in vivo* loading of mouse right tibia knee joint, but proved to work similarly for both left and right legs. The same cups were previously used for investigating strains on the tibia of 8 w.o., female, B6 mice (Sztefek et al., 2010). When comparing the results, we observed a decrease of the strain magnitude on the bone surface of 12 w.o. mouse under similar loading conditions (i.e. with only the femur attached). This probably indicates changes in normal bone morphology that occur between 8 and 12 w.o., and/or between male and female mice, but it can also be due to a different rotation of the bone in front of cameras and/or to different preserving methods used for these studies: we used fresh frozen bones while the bone used in previous studies (Sztefek et al., 2010) were rehydrated from ethanol preservation.

### 4.1 Conclusion

This work provides a better understanding of parameters affecting full field strain measurements from DIC in *ex vivo* murine tibial loading tests. The heterogeneity of the bone surface determines local deformations and strains, which are captured by tracking the displacements of unique material points on the surface at high resolution but cannot be captured using strain gauges or approximate finite element models. Revealing local microstructural strains in cortical bone is not only essential for enhancing our understanding of bone adaptation, but can also provide insights in other fields, such as bone damage development and fracture.

## Conflict of interest statement

The authors have no conflict of interest.

## Figures and Tables

**Table 1:** Set up tests and results of the strain measurements with DIC for *ex vivo* loading tibia.

	**Methods** (C57BL/6 mice, both legs, male, 18 w.o.)	**Results**
**Speckle distribution** (tibiae from 3 mice)	• *Speckle 1*: ∅ =~4 pixels and 40% black density • *Speckle 2*: ∅ =~8 pixels and 45% black density • *Speckle 3*: ∅ =~12 pixels and 55% black density	A speckle size of ~8 pixels diameter and 45% black density is desirable when using a 19×19 pixels facet size. High density increases the maximum strain computed on bone.
**Facet size and overlapping** (tibiae from 3 mice)	• 15× 15 pixels with 7% overlap • 19×19 pixels with 20% overlap • 20×20 pixels with 5% overlap	Smaller facet sizes may create problems in computing a strain map if the speckle is as big as the facet size
**Right and left leg** (tibiae from 6 mice)		loading cups work similarly for both leg sides
**Precision of measurement on a single bone**	10 tests of same bone with same speckle	Strain maps using same bone and speckle are comparable
**Loading positions within the cups** (tibiae from 3 mice)	• *Position 1*: tibia loaded axially • *Position 2*: tibia loaded at a 30° to the vertical axis	Tibia loaded in *Position 2* self-aligns to *Position 1* while loaded up to 12 N
**Rotations in front of the cameras** (tibiae from 2 mice)	• *Rotation 1*: medial surface parallel to the camera axis • *Rotation 2*: medial surface at −45° to the camera axis • *Rotation 3*: medial surface at +45° to the camera axis	• Bone surface of interest must be parallel to the camera axis • If comparing strain maps, orient the bones similarly
**Loading configurations** (tibiae from 3 mice, 12 w.o.)	The tibia was loaded with: • The full mouse body • the foot and femur with its muscles • only the foot	• Strain magnitude may further change if tibial muscles are attached, but DIC requires the surface to be exposed. • If strain magnitudes are required, it is important to closely replicate the loading conditions *in vivo*. If strain patterns are more of interest, comparisons can still be made across bones with a consistent tibia-femur set-up.
